# Postoperative Complications Following Open Reduction and Internal Fixation of Mandibular Condylar Fractures Using the High Perimandibular Approach: A Multicenter Retrospective Study

**DOI:** 10.3390/cmtr18040047

**Published:** 2025-10-25

**Authors:** Noriko Sakata, Masako Fujioka-Kobayashi, Yuhei Matsuda, Reon Morioka, Erina Toda, Shinji Ishizuka, Michitaka Somoto, Rie Sonoyama-Osako, Hiroto Tatsumi, Takahiro Kanno

**Affiliations:** 1 Department of Oral and Maxillofacial Surgery, Shimane University Faculty of Medicine, 89-1, Enya-Cho, Izumo 693-8501, Shimane, Japan; n.sakata@med.shimane-u.ac.jp (N.S.); masako@med.shimane-u.ac.jp (M.F.-K.); yuhei@med.shimane-u.ac.jp (Y.M.); moriokareon@med.shimane-u.ac.jp (R.M.); et1211@med.shimane-u.ac.jp (E.T.); ishizuka@med.shimane-u.ac.jp (S.I.); somoto.m@med.shimane-u.ac.jp (M.S.); r.osako@med.shimane-u.ac.jp (R.S.-O.); tatsumi@med.shimane-u.ac.jp (H.T.); 2 Department of Pharmacology, Shimane University Faculty of Medicine, 89-1, Enya-Cho, Izumo 693-8501, Shimane, Japan; 3 Department of Oral and Maxillofacial Surgery, National Hospital Organization Hamada Medical Center, 777-12, Asai-Cho, Hamada 697-0022, Shimane, Japan; 4 Department of Oral and Maxillofacial Surgery, Masuda Red Cross Hospital, 103-1, Otoyoshi-Cho, Masuda 698-0003, Shimane, Japan; 5 Department of Medical Oncology, Shimane University Hospital, 89-1, Enya-Cho, Izumo 693-8501, Shimane, Japan

**Keywords:** high perimandibular approach, mandibular condylar fracture, trismus, masseter muscle

## Abstract

Background: The high perimandibular approach (HPA) is a feasible surgical technique for open reduction and internal fixation (OR-IF) of mandibular condylar fractures, offering reduced complication rates. In this study, we retrospectively evaluated the treatment outcomes and complications associated with HPA use. Patients and Methods: Patients who underwent OR-IF for mandibular condylar fractures using the HPA at three hospitals in Shimane between June 2019 and March 2024 were included. Data collected included the mechanism of injury, AO classification of the fracture site, fracture type and mode, surgical duration, mouth-opening range at 6 months post-operatively, and peri- and post-operative complications. Results: A total of 42 patients (46 condylar fractures; 18 males and 24 females; mean age, 63.0 years) were included. The fracture pattern included dislocations in 18 cases (42.8%). The mean surgical duration was 75.0 min. Post-operative trismus occurred in 16 patients (38.1%) at 6 months. Longer surgical duration and dislocated fractures were significantly associated with post-operative trismus (*p* < 0.05). Conclusions: The HPA is safe and effective for managing mandibular condylar fractures. However, post-operative trismus may be influenced by longer surgical duration and fracture types, warranting further investigation and potential post-surgical management.

## 1. Introduction

Condylar fractures are the most common mandibular fractures, accounting for approximately 19–52% of cases [1,2,3,4]. Condylar fractures usually occur indirectly when external forces are applied to the chin or parasymphysis and transmitted to the condyle. Less commonly, they may result from direct trauma to the condylar region itself. Fractures occur in the subcondylar, neck, and head regions with reported incidences of 18–70%, 18–53%, and 9–54%, respectively [3,4,5,6]. Fracture types include deviation, displacement, deviated dislocation, and displaced dislocation, which occur in 12–82%, 18–69%, 8%, and 44% of cases [3,4,5,6]. Treatment selection should be based on the fracture site and type to optimize outcomes.

Condylar fractures are treated either conservatively or with open reduction and internal fixation (OR-IF). Conservative therapy, typically with intermaxillary fixation (IMF), is suitable for children and minimally displaced fractures but carries risks of malocclusion, joint issues, and esthetic problems [7]. OR-IF is preferred for displaced fractures or when functional and esthetic restoration is required, as it enables precise fixation and faster recovery. A meta-analysis revealed that OR-IF in adults resulted in reduced post-operative malocclusion and improved function compared with that using conservative treatment [8]. However, OR-IF also has disadvantages, including the possibility of facial nerve damage, scar formation, and post-operative infection; therefore, the chosen surgical procedure and the surgeon’s skill affect treatment outcomes.

OR-IF for mandibular condylar fractures can be performed using the Risdon, preauricular, retromandibular, transmasseteric anteroparotid (TMAP), transoral endoscopic, and high perimandibular (HPA) approaches [9,10,11]. Each approach is associated with a risk of post-operative complications corresponding to the anatomical characteristics; therefore, understanding the characteristics of each surgical technique is important.

The Risdon approach is a classical technique for OR-IF in mandibular condylar fractures and is characterized by access from the lower border of the mandible below the marginal mandibular branch of the facial nerve to reach the fracture site [12]. While it is widely used, it provides insufficient exposure of the mandibular ramus and condyle, with reported facial nerve palsy rates of 4.2–27.3% [12,13]. The preauricular approach is often used for high mandibular condylar fractures, such as intracapsular condylar fractures [14,15]; however, it is unsuitable for low condylar fractures because of limitations in exposing the mandibular angle [15]. Complications associated with the preauricular approach include scarring, sensory loss, and Frey syndrome [15]. Additionally, the average maximum mouth opening after the preauricular approach is 34.6 mm, and the incidence of facial nerve damage is 17.5% [16]. The retromandibular approach provides a clear view of the entire fracture site from the posterior border to the condylar process, and allows access directly from above the fracture site, making it a highly maneuverable technique for obtaining a clear surgical field [17,18]. However, post-operative complications include temporary or permanent facial paralysis (17.2%) [17,19], infection (11.9%), salivary fistulas (3.4%), sialocele (1.7%), Frey syndrome (0.8%), and cosmetically undesirable scarring (7.5%) [17]. The median mouth opening at 6 months post-operatively is 42.5 mm [18].

TMAP is advantageous as it provides a clear surgical view during the reduction and fixation of subcondylar fractures [10]. Furthermore, TMAP is associated with low facial nerve palsy rates and is particularly useful in dislocation fractures in female patients [20]. Reported post-operative complication rates include salivary fistula (0%), sialocele (0–4.5%), non-conforming scar (4.5%), infection (0–6.0%), masseter muscle pain (6.8%), malocclusion (9%), plate fracture/displacement (0%), and facial nerve palsy (0–6.8%) [10,11,13,20,21]. A reduced maximal mouth opening (<37 mm) occurs in 6.7% of patients within 6–12 months post-operatively [11]. The transoral endoscopic approach is performed solely through oral manipulation to minimize the possibility of damage to the facial nerve. However, it also has limitations, including prolonged surgical duration and restricted surgical site visibility, and is only applicable to low condylar neck fractures with minimal displacement [17,18]. The median mouth opening 6 months post-operatively is 43 mm [18].

The HPA, a surgical procedure first described by Wilk et al. in 1997 [22], is indicated for accessing the condylar neck and subcondylar region. A 2018 systematic review and meta-analysis reported that the HPA carries the lowest risk of facial nerve damage [20]. It is characterized by a higher incision line than that of the submandibular or retromandibular approaches, allowing access closer to the fracture site [23]. The HPA is performed from a more cranial position than the marginal mandibular branch of the facial nerve, making exposure of this branch intra-operatively less likely and reducing the amount of tissue traction required to expand the surgical field. Consequently, the incidence of facial nerve damage is significantly lower than that with other approaches. Imai et al. reported that deep approaches, such as the traditional submandibular and retroparotid approaches (performed from below the marginal mandibular branch), carry a higher risk of post-operative facial nerve damage than that of superficial approaches, such as TMAP and HPA (performed from above the marginal mandibular branch) [20]. Additionally, the HPA incision line is hidden by the mandibular angle, resulting in less visible scarring.

On the other hand, HPA may also be associated with postoperative mouth opening disorders [23]. A single-center retrospective cohort study published in 2023 on post-operative complications associated with mandibular condylar fracture surgery using the HPA in our department showed that 15% of patients developed mild opening disorders (<35 mm maximum self-reported mouth-opening distance at 6 months post-operatively), which did not interfere with daily life [24]. That study also showed that the fracture site and surgery duration are significant risk factors for reduced post-operative mouth opening, and that damage to the masseter muscle and surrounding tissues is a risk factor [24].

Despite these findings, few studies have been conducted to explore the risk factors for the occurrence of post-operative complications after HPA. Therefore, we conducted a multicenter study to investigate the risk factors for post-operative complications after HPA and improve the safety and functional outcomes of the procedures. In particular, we aimed to clarify factors contributing to the onset of post-operative mouth opening disorders, including patient characteristics, fracture patterns, surgeon-related variables, and facility-related factors. The results of this study will contribute to the improvement in HPA surgical techniques and post-operative management methods.

## 2. Patients and Methods

### 2.1. Patient Eligibility

In this multicenter retrospective study, we used data from patients with mandibular condylar neck or subcondylar fractures treated at three hospitals in Shimane Prefecture: Shimane University Hospital (Department of Oral and Maxillofacial Surgery/Maxillofacial Trauma Center), Hamada Medical Center (Department of Oral and Maxillofacial Surgery), and Masuda Red Cross Hospital (Department of Oral and Maxillofacial Surgery). Patients with mandibular condylar fractures treated between June 2019 and September 2023 were included in this study. Patient inclusion criteria were as follows: OR-IF of the neck or subcondylar fracture using HPA, and completion of adequate imaging and clinical prognostic evaluation for ≥6 months. Exclusion criteria included cases with missing data and patients who refused to allow their data to be used.

This study was approved by the Medical Research Ethics Committee of Shimane University Faculty of Medicine (approval number: KS20240506-3). All procedures were performed in accordance with the principles outlined in the Declaration of Helsinki.

### 2.2. Collected Data

#### 2.2.1. Patient Background

The following baseline data were collected from the electronic medical records of the participating hospitals: sex (male/female), age (in years), and body mass index (in kg/m^2^).

#### 2.2.2. Data on Fracture and Open Reduction and Internal Fixation

Collected variables included the cause of fracture, fracture site (AO classification) [25], fracture type (MacLennan classification) [26], presence of complicated maxillofacial fractures, surgical duration for mandibular condylar fractures only (duration from incision to suturing for mandibular condylar fractures alone), and surgeon experience. Surgical duration data for mandibular condylar fractures alone were obtained from electronic medical records accurately recorded by anesthesiologists.

#### 2.2.3. Data on Complications

Post-operative complication data were collected from medical records. Post-operative follow-up was conducted as follows: During hospitalization (with detailed observation for 10 days post-operatively), patients without concomitant injuries or complications, except for those involving the maxillofacial region, were evaluated during outpatient visits at 1, 3, and 6 months post-operatively, and patients with post-operative complications were scheduled for additional visits. Imaging evaluations were performed on the day after surgery and at 1 and 6 months post-operatively. Surgical site infection was defined as pus discharge from the wound or incomplete wound healing. Facial nerve palsy was evaluated based on the House–Brackmann method, assessing forehead wrinkling, ability to close the eyes, and mouth drooping [27]; classified as temporary if it healed within 6 months post-operatively and permanent if it did not heal. A salivary fistula was defined as a tract with serous exudate from the wound site and no bacteria detected on microscopic examination. Malocclusion was defined as a subjective complaint of occlusal discomfort. Temporomandibular joint pain was defined as patient-reported temporomandibular joint pain at rest or during movement at 6 months post-operatively. A poor state of reduction was defined as the presence of abnormalities in the anatomical position of the reduced bone fragments on computed tomography (CT) 6 months post-operatively. Surgical scar perceptibility was defined as thickening or redness of the wound based on the patient’s subjective assessment and the physician’s evaluation. Plate breakage or screw loosening was defined as plate breakage or screw loosening identified on CT images taken 6 months post-operatively. Trismus was defined as the maximum self-reported mouth-opening distance at 6 months post-operatively of less than 40 mm. The cutoff value of 40 mm for the amount of mouth opening is based on the guidelines of the Japan Prosthodontic Society [28]. This 40 mm value was determined considering Japanese lifestyle habits and dietary customs related to oral health.

#### 2.2.4. Data Obtained via Computed Tomography Evaluation

**Figure 1:**
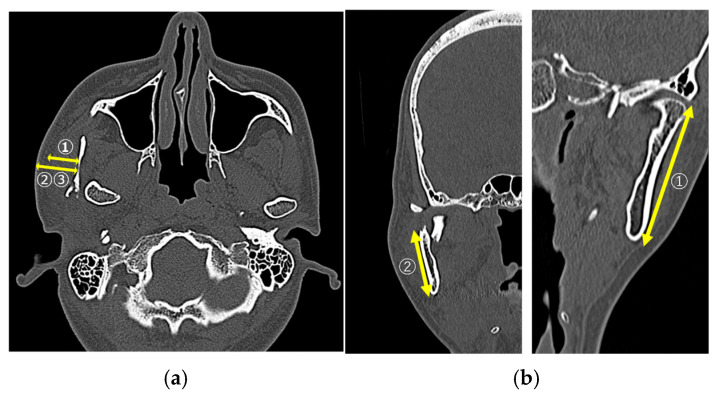
(**a①**) Masseter muscle thickness; (**a②**) skin-to-fracture distance line (at the level of the mandibular notch; (**a③**) skin-to-fracture distance line (at the level of the mandibular foramen); (**b①**) the mandibular ramus length; (**b②**) the distance from the mandibular border to the center of the fracture line.

In the CT image evaluation, measurements were obtained on the fractured side for all items. The masseter muscle thickness was measured at the mandibular foramen level on the axial images (Figure 1a①). The skin-to-fracture distance line was measured at the level of the mandibular notch (mm, Figure 1a②) and the mandibular foramen (mm) on axial images (Figure 1a③). Mandibular ramus length (mm) was measured as the distance from the inferior border of the mandible to the mandibular condyle on coronal images (Figure 1b①). The distance from the inferior border of the mandible to the center of the fracture line (mm) was measured as a straight-line distance on coronal images (Figure 1b②).

### 2.3. Statistical Analysis

The Shapiro–Wilk test was used to confirm the normality of the data distribution. Continuous data were described as mean ± standard deviation or as medians with interquartile ranges (25th–75th percentile), while categorical data were described as numbers and percentages. Intergroup comparisons were performed using the chi-square and Mann–Whitney U-tests. Multiple imputations were used to identify missing values. Statistical analyses were performed using IBM SPSS Statistics for Windows version 29 (IBM Corp., Armonk, NY, USA). Two-tailed *p*-values were calculated for all analyses, and the alpha level of significance was set at *p* < 0.05.

### 2.4. Surgical Procedure (High Perimandibular Approach)

**Figure 2:**
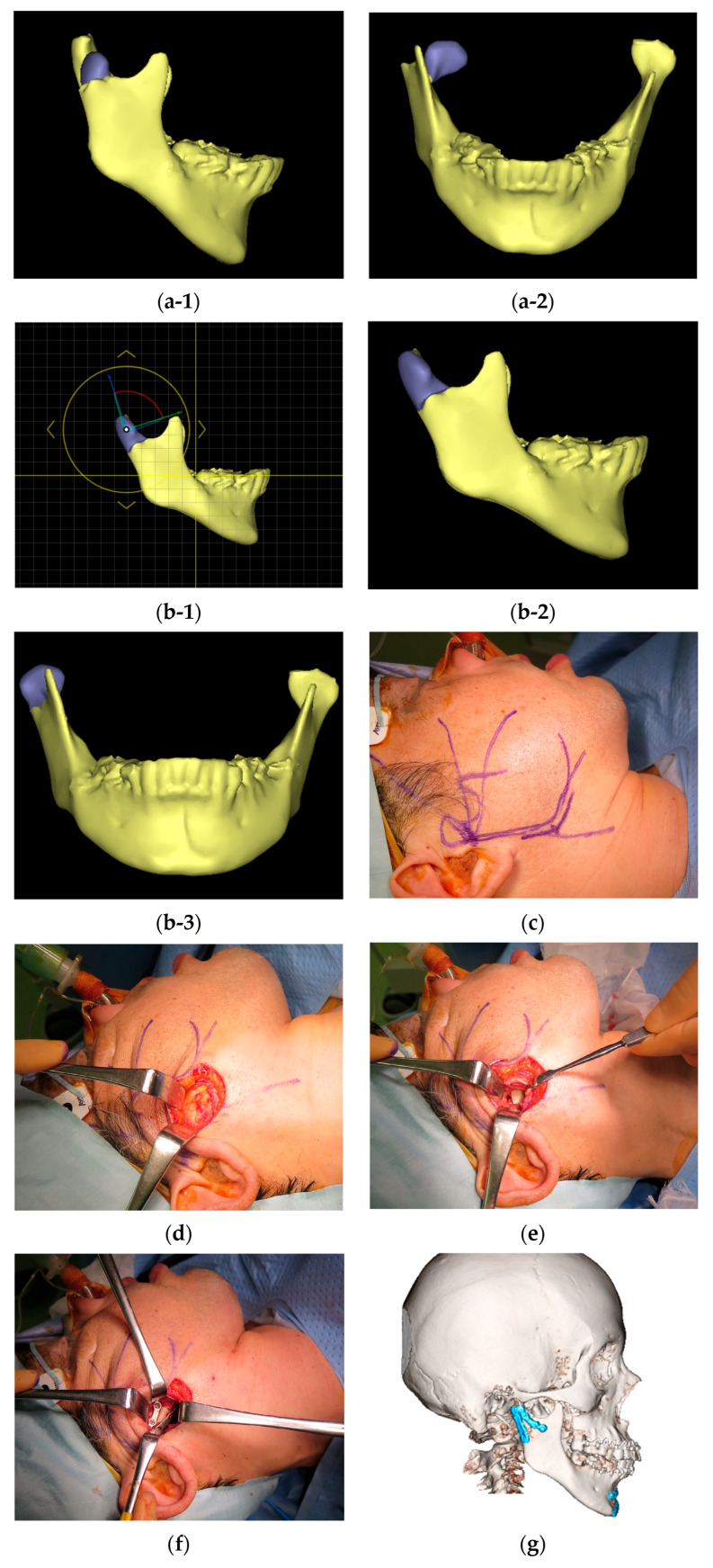
(**a**) Preoperative images of the mandible were generated using computed tomography (CT) data with a simulation software (Enlight CMF 6.0, Materialise Co., Ltd., Yokohama, Japan: (**a-1**) right lateral view; (**a-2**) frontal view. (**b**) Reduction in bone fragments on the simulation software, taking into account anatomical relationships: (**b-1**) determination of reduction position on the simulation software; (**b-2**) planned reduction position on the right lateral view; (**b-3**) planned reduction position on the frontal view. (**c**) Skin incision. (**d**) Removal of the platysma muscle to expose the masseter muscle. (**e**) After incising the masseter muscle vertically along the masseter muscle fibers, the masseter muscle was detached from the mandible along with the periosteum to expose the fracture line. (**f**) Two buttresses were rigidly fixed with a titanium plate. (**g**) A CT scan was taken the day after surgery, confirming that proper reduction and fixation had been achieved.

Diagnosis and treatment planning were performed using three-dimensional computer simulations. The surgeon discussed the simulation results with the supervising physician to establish a diagnosis and treatment plan (Figure 2a-1,a-2,b-1–b-3). The basic HPA surgical procedure and postoperative management included the following steps: IMF was performed using screws and a 0.5 mm wire to immobilize the maxilla and mandible in centric occlusion. A 4–5 cm incision line was made 0.5 cm below the lower border of the mandible (Figure 2c). After skin incision, the platysma muscle was adequately exposed (Figure 2d). A 1 cm incision was made parallel to the lower border of the mandible. Care was taken to avoid damage to the facial nerve; the platysma muscle was undermined, and the masseter fascia was exposed. The masseter fascia was incised by making an incision perpendicular to the masseter fibers to reach the mandibular bone surface, and the masseter muscle was peeled along with the periosteum from the mandible to expose the fracture site (especially in cases of dislocation of bone fragments, an IMF screw was placed in the distal bone fragment, which was pulled downward to create some space, and a reduction technique was used for the dislocated bone fragment; Figure 2e). Two buttresses were fixed at the mandibular notch and the posterior margin of the mandibular ramus using a titanium plate (Figure 2f,g). The IMF was subsequently removed, the absence of issues with mandibular movement was confirmed, and the wound was closed. Post-operatively, IMF was not performed, and only occlusal guidance using elastic rubbers was performed.

## 3. Results

### 3.1. Patient Background

A total of 42 patients with 46 condylar fractures were enrolled, including 18 (42.9%) males and 24 (57.1%) females, with a median age of 63 years. The background factors and information about mandibular condylar fractures and complications are summarized in Table 1.

### 3.2. Data on Fractures and Treatment

Of the 42 patients, 6 (14.3%) were classified as having a high neck, 15 (35.7%) as having a low neck, and 21 (50.0%) as having a subcondylar neck. Table 2 shows detailed data on fractures and treatment.

### 3.3. Data on Complications

A total of 19 patients (45.2%) developed complications, with opening disorders being the most common complication (Table 3).

### 3.4. Exploratory Analysis of Risk Factors for Complications

Table 4 shows the results of group comparisons between the trismus (16 patients [38.1%]) and the non-trismus (26 patients [61.9%]) groups for patients’ background and clinical data. No significant differences were observed between groups in terms of complications other than trismus.

### 3.5. Sub-Analysis of Dislocation

As an additional exploratory sub-analysis of dislocation, Table 5 shows group comparisons of factors associated with dislocation.

## 4. Discussion

Two major findings were identified in this study. First, dislocation-type fractures were associated with trismus 6 months post-operatively. In such cases, anatomical reduction in the bone fragments is difficult, often requiring more extensive soft tissue dissection and traction [29]. It is necessary to create a three-dimensional working space to reduce the bone fragments. In our department, we create a space by inserting screws into the distal bone fragment and pulling it downward. With the HPA for mandibular condylar fractures, the average mouth opening 1 month post-operatively is 36.4 mm (standard deviation 1.95), with approximately 96.8% of patients experiencing trismus of 40 mm or less. Six months after surgery, the average mouth opening is 40.4 mm (standard deviation 1.17), with approximately 36.7% experiencing trismus of 40 mm or less [30]. The extent of mouth opening observed in this study was 38.1%, which is consistent with the results of the previous study. In the same report, the average mouth opening after the retromandibular approach was 39.9 mm (standard deviation, 1.37) at 6 months, with approximately 52.9% of the patients experiencing trismus. This shows that the HPA is not particularly associated with trismus [30]. Trismus is caused by tumor invasion in the oral/head and neck area, organic changes in the tissue due to surgery or radiation therapy for tumors, inflammation, trauma, and congenital abnormalities in the oral and maxillofacial regions. Muscles associated with trismus include the masseter and lateral and medial pterygoids [31,32,33]. In particular, masseter plays an important role in mouth opening movements, and dysfunction of the masseter can cause trismus [34]. Surgery in the masticatory muscle region can cause trismus [35]. Kim et al. reported that post-operative swelling caused by bleeding within the muscle, muscle atrophy, and scar formation due to masseter muscle resection can cause short- or long-term trismus [36]. In this study, the amount of mouth opening was evaluated 6 months after treatment; however, a one-year follow-up of trismus caused by oral cancer treatment with surgery alone showed that the amount of mouth opening often improves after one year [37]. In particular, trismus caused by surgery is more likely to recover during long-term follow-up and rehabilitation than trismus caused by radiation therapy [37,38]. Therefore, further follow-up of patients with HPA may reveal improvements in mouth opening. In addition, the cut-off value for determining trismus in this study was set at 40 mm, which is the standard value determined by the Japan Prosthodontic Society [28]. This cutoff value of 40 mm is stricter than the 35 mm criterion reported for trismus in other diseases; therefore, the impact of HPA-induced trismus on daily life and quality of life may have been smaller.

The second major finding was that prolonged surgical duration for reduction in dislocation fracture may be associated with an increased risk of trismus. In this study, despite the assumption that individual physical characteristics and anatomical differences (such as masseter muscle and skin thicknesses) are associated with post-operative complications, no significant correlations were found. This result suggests that muscle and skin thicknesses have no significant effect on the difficulty in reaching the surgical site or the degree of invasiveness during surgery. However, the procedure of reducing and fixing the dislocated bone fragments is more closely related to tissue damage than to the distance to the fracture site. The HPA is designed to avoid the course of the mandibular branch of the facial nerve, thus contributing to a reduction in the risk of nerve damage. However, other surgical factors (particularly the invasion of muscle fibers and surrounding tissues) may cause trismus [39]. Therefore, sufficient care must be taken during surgical procedures, especially in cases of dislocation [36,39]. In the future, to reduce post-operative trismus, improvements will be required from a clinical perspective in intraoperative techniques for dislocation, reduction in surgical duration, and techniques for tractioning tissue. For example, the development of devices that simplify the reduction in dislocated bone fragments and improvements in rehabilitation techniques, such as early postoperative mouth-opening exercises, may contribute to reducing trismus.

As a minor finding, plate loosening occurred in 4.8% of cases, which is similar to the incidence rate of 2.9% reported in previous studies. In two cases, the screws appeared loose on imaging, but the bone fragments healed while maintaining their anatomical shape, and no additional surgery was needed. Because the incidence rate is extremely low, the HPA efficacy is not considered impaired [40]. Salivary fistula occurred in only one case (2.4%), similar to the incidence rates reported previously [30,41]. The low incidence of salivary fistula could be because transecting the masseter muscle from the supraplatysmal layer in the HPA reduces the risk of damage to the parotid gland and the subsequent development of Frey syndrome [23]. In cases of subcondylar fractures accompanied by severe lateral displacement of bone fragments, damage to the parotid duct and the development of salivary fistulas have been reported, regardless of the surgical approach, suggesting that salivary fistulas are not a complication specific to the HPA [42]. In this study, no cases of facial nerve palsy were observed, which is consistent with the extremely low incidence rate of 0–0.9% reported in previous studies [13,41,43,44]. In contrast, in the retromandibular transparotid approach, subcondylar fractures, dislocations, and lack of surgeon experience are significantly related to the occurrence of facial nerve palsy after surgery [45]. In this study, there was no correlation between post-operative complications and the postgraduate year of the surgeon while performing the HPA, suggesting that it is an easy and safe approach with few surgeon-dependent risk factors. There were no cases of surgical site infection, temporomandibular joint pain, facial nerve palsy, or scar formation at the surgical site. The salivary fistula also healed with 5 days of gauze compression and was not a serious complication. Based on the above findings, trismus is not a significant concern associated with the HPA compared to that with the other approaches, and HPA can be considered relatively safe, with few complications and easy surgical procedures.

This study has three limitations: 1. Since the follow-up period for trismus was only 6 months, more detailed research is needed with long-term follow-up and the impact of trismus on daily life. 2. Because no direct comparison with other surgical procedures was conducted, evidence demonstrating the superiority of HPA is limited. 3. This study was limited to Japanese patients treated at three facilities in the Shimane Prefecture. In the future, international multi-center studies including prospective cohort studies and randomized controlled trials should be conducted to examine skeletal differences between races.

## 5. Conclusions

Although mouth opening disorders are the most common complications of the HPA, it is considered safe because condylar dislocation is a more severe complication than a surgeon-dependent factor.

## Figures and Tables

**Table 1:** Demographic characteristics of patients with mandibular condylar fractures (*n* = 42).

Variables	Categories	*n* (%), Median [25–75%]
Sex	Male	18 (42.9)
	Female	24 (57.1)
Age (years)		63.0 [35.0–79.3]
Body Mass Index (kg/m^2^)		20.7 [17.9–24.3]

**Table 2:** Descriptive data on fracture and open reduction and internal fixation.

Variables	Categories		*n* (%), Median [25–75%]
Cause of injury	Slip		26 (61.9)
	Traffic accident		6 (14.7)
	Sports		2 (4.8)
	Violence		2 (4.8)
	Work		2 (4.8)
	Fall		4 (9.5)
Site of fracture	High neck		6 (14.3)
	Low neck		15 (35.7)
	Subcondylar		21 (50.0)
Type of fracture	Deviation		20 (47.6)
	Displacement		4 (9.5)
	Dislocation	Deviated dislocation	9 (21.4)
		Displaced dislocation	9 (21.4)
Maxillofacial fractures other than mandibular condylar fractures	Mandible		19 (45.2)
	Midface		1 (2.4)
	Mandible and midface		3 (7.1)
High-energy trauma	Yes		7 (16.7)
Operation time (min)			75.0 [50.5–89.0]
Postgraduate year of the surgeon			5.0 [5.0–5.0]

**Table 3:** Descriptive data on complications.

Variables	Categories	*n* (%), Median [25–75%]
Amount of mouth opening at 6 months postoperatively		40.0 [36.5–45.0]
The number of complications of fractures		1.0 [0.0–1.0]
Complications	Facial nerve palsy (temporary)	0 (0)
	Facial nerve palsy (permanent)	0 (0)
	Poor state of reduction	0 (0)
	Surgical site infection	0 (0)
	Postoperative malocclusion	0 (0)
	Salivary fistula (parotid fistula)	1 (2.4)
	Plate breakage or screw loosening	2 (4.8)
	Surgical scar perceptibility	0 (0)
	Temporomandibular joint pain	0 (0)
	Trismus at 6 months postoperatively	16 (38.1)

**Table 4:** Group comparison between the trismus and non-trismus groups.

Variables	Categories	*n* (%), Median [25–75%]		*p*-Value
		Trismus Group (*n* = 16)	Non-Trismus Group (*n* = 26)	
Sex	Male	5 (31.3)	13 (50.0)	0.12 ^a^
	Female	11 (68.8)	13 (50.0)	
Age (years)		64.0 [35.8–83.0]	62.5 [33.5–79.3]	0.77 ^b^
Body Mass Index (kg/m^2^)		20.1 [16.5–25.2]	20.7 [18.4–23.3]	0.46 ^b^
Cause of injury	Slip	10 (62.5)	16 (61.5)	0.87 ^a^
	Traffic accident	3 (18.8)	3 (11.5)	
	Sports	1 (6.3)	1 (3.8)	
	Violence	0 (0)	2 (7.7)	
	Work	1 (6.3)	1 (3.8)	
	Fall	1 (6.3)	3 (11.5)	
Site of fracture	High neck	7 (43.8)	4 (15.4)	0.60 ^a^
	Low neck	7 (43.8)	8 (30.8)	
	Subcondylar	2 (12.5)	14 (53.8)	
	Condylar neck	14 (87.5)	12 (46.2)	0.75 ^a^
	Condylar base	2 (12.5)	14 (53.8)	
Type of fracture	Deviation	4 (25.0)	16 (61.5)	0.06 ^a^
	Displacement	1 (6.3)	3 (11.5)	
	Deviated dislocation	6 (37.5)	3 (11.5)	
	Displaced dislocation	5 (31.3)	4 (15.4)	
	Dislocation (yes)	11 (68.8)	7 (26.9)	0.02 ^a^*
Maxillofacial fractures other than mandibular condylar fractures	Mandible	5 (31.3)	13 (50.0)	0.35 ^a^
	Midface	1 (6.3)	1 (3.8)	
	Mandible and midface	1 (6.3)	2 (7.7)	
High-energy trauma	Yes	4 (25.0)	3 (11.5)	0.49 ^a^
Operation time (min)		85.0 [65.0–97.8]	58.0 [49.0–78.8]	0.02 ^b^*
Postgraduate year of the surgeon		9.0 [4.0–16.0]	8.0 [3.8–15.3]	0.25 ^b^
Presence of associated maxillofacial fractures	Yes	7 (43.8)	16 (61.5)	0.14 ^a^
The number of complications of the fracture		0.0 [0.0–1.0]	1.0 [0.0–1.0]	0.22 ^b^
Thickness of the masseter muscle		13.6 [11.2–15.1]	14.1 [11.7–17.0]	0.51 ^b^
Skin thickness at the level of mandibular notch		24.0 [19.6–27.5]	24.4 [21.3–26.8]	0.57 ^b^
Skin thickness at the level of the mandibular foramen		23.0 [20.5–27.0]	22.5 [17.2–24.9]	0.88 ^b^
Length from the inferior border of the mandible to the fracture line		39.9 [35.2–44.4]	40.2 [35.5–46.1]	0.87 ^a^
Mandibular ramus height		60.0 [57.3–67.6]	62.0 [55.8–68.9]	0.50 ^b^

^a^: chi-square test; ^b^: Mann–Whitney U-tests; *: *p* < 0.05.

**Table 5:** Group comparison between the dislocation and non-dislocation groups.

Variables	*n* (%), Median [25–75%]		*p*-Value
	Dislocation Group (*n* = 23)	Non-Dislocation Group (*n* = 19)	
Operation time (min)	81.0 [76.0–96.0]	51.0 [47.0–79.0]	0.01 ^a^*
Range of mouth opening at 6 months postoperatively	38.0 [35.0–42.0]	42.0 [40.0–45.0]	0.04 ^a^*

^a^: chi-square test; *: *p* < 0.05.

## Data Availability

The data supporting the findings of this study are not publicly available because the study protocol did not include a provision for publicly shared data. Approval must be obtained from the Medical Research Ethics Committee of Shimane University Faculty of Medicine (kenkyu@med.shimane-u.ac.jp) to request the provision of de-identified data.

## Abbreviations

The following abbreviations are used in this manuscript:

HPA	High Perimandibular Approach
IMF	Intermaxillary Fixation
OR-IF	Open Reduction and Internal Fixation
TMAP	TransMasseteric AnteroParotid approach
